# Revision history aware repositories of computational models of biological systems

**DOI:** 10.1186/1471-2105-12-22

**Published:** 2011-01-14

**Authors:** Andrew K Miller, Tommy Yu, Randall Britten, Mike T Cooling, James Lawson, Dougal Cowan, Alan Garny, Matt DB Halstead, Peter J Hunter, David P Nickerson, Geo Nunns, Sarala M Wimalaratne, Poul M F Nielsen

**Affiliations:** 1 Auckland Bioengineering Institute, The University of Auckland, Private Bag 92019, Auckland, NZ; 2 Department of Physiology, Anatomy and Genetics, University of Oxford, Sherrington Building, Parks Road, Oxford OX1 3PT, UK; 3 Johns Hopkins University, Baltimore, Maryland, USA; 4 EMBL Outstation, European Bioinformatics Institute, Wellcome Trust Genome Campus, Hinxton, Cambridge CB10 1SD, UK; 5 Department of Engineering Science, Faculty of Engineering, The University of Auckland, Private Bag 92019, Auckland, NZ

## Abstract

**Background:**

Building repositories of computational models of biological systems ensures that published models are available for both education and further research, and can provide a source of smaller, previously verified models to integrate into a larger model.

One problem with earlier repositories has been the limitations in facilities to record the revision history of models. Often, these facilities are limited to a linear series of versions which were deposited in the repository. This is problematic for several reasons. Firstly, there are many instances in the history of biological systems modelling where an 'ancestral' model is modified by different groups to create many different models. With a linear series of versions, if the changes made to one model are merged into another model, the merge appears as a single item in the history. This hides useful revision history information, and also makes further merges much more difficult, as there is no record of which changes have or have not already been merged. In addition, a long series of individual changes made outside of the repository are also all merged into a single revision when they are put back into the repository, making it difficult to separate out individual changes. Furthermore, many earlier repositories only retain the revision history of individual files, rather than of a group of files. This is an important limitation to overcome, because some types of models, such as CellML 1.1 models, can be developed as a collection of modules, each in a separate file.

The need for revision history is widely recognised for computer software, and a lot of work has gone into developing version control systems and distributed version control systems (DVCSs) for tracking the revision history. However, to date, there has been no published research on how DVCSs can be applied to repositories of computational models of biological systems.

**Results:**

We have extended the Physiome Model Repository software to be fully revision history aware, by building it on top of Mercurial, an existing DVCS. We have demonstrated the utility of this approach, when used in conjunction with the model composition facilities in CellML, to build and understand more complex models. We have also demonstrated the ability of the repository software to present version history to casual users over the web, and to highlight specific versions which are likely to be useful to users.

**Conclusions:**

Providing facilities for maintaining and using revision history information is an important part of building a useful repository of computational models, as this information is useful both for understanding the source of and justification for parts of a model, and to facilitate automated processes such as merges. The availability of fully revision history aware repositories, and associated tools, will therefore be of significant benefit to the community.

## Background

Building mathematical models of biological systems plays an important role in understanding those systems. Early work on building models for computational simulation conflated the model with the techniques to simulate it, and used procedural programming languages. The mathematical equations in the model were published, rather than the computational code, and so reproducing the simulation results involved a significant amount of work.

More recently, declarative formats for marking up mathematical models have been developed. CellML [1] and SBML [2], for example, both use an XML based format to represent models. The focus of CellML is on representing mathematical models, while SBML contains constructs specific to modelling reaction networks, but allows certain additional forms of algebraic and rate equations. The benefit of these declarative representations is that the numerical algorithm to be used is completely separated from the mathematical specification of the model. This means that the same model description can be used to solve the model with many different tools and numerical algorithms. It also means that all or parts of the model can be more easily taken and put into a different model.

The benefit of having models in a few widely used standard formats is that the tools needed to run simulations are gradually becoming ubiquitous within the biological systems modelling community, or at least readily available. There are well-developed APIs to simplify the development of tools for working with CellML [3] and SBML [4] models. In addition, model users can choose from a wide range of programs for running analyses on CellML [5] and SBML [6-9] models. Thus, modellers can share their models with members of the community, who can not only easily reproduce the results described in a paper, but also build on the work of other modellers.

This benefit becomes even more significant when common parts of models can be shared between multiple models. CellML 1.1 provides for model composition by allowing hierarchies of components to be imported from one model into another, where they can be connected to other components. For example, CellML model composition has been used to build a library of models for standard synthetic biology parts [10].

**Figure 1:**
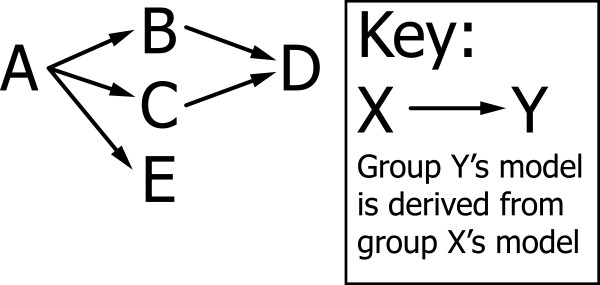
**Example of a hypothetical revision history**. This figure demonstrates the revision history of a hypothetical model, and shows the non-hierarchical nature of the revision history graph.

In order for model sharing to occur, it is important that members of the community can easily obtain models. A number of repositories have been developed for sharing models, including the Physiome Model Repository 2 (PMR2) software (which is used to run the CellML Model Repository [11]) and the BioModels Database [12]. No repository described in the literature to date has had the ability to store detailed information on the revision history of the representation of the model. Figure 1 gives a hypothetical example of the level of complexity of revision histories. In this example, group A created a model of a particular cell type. Group B took group A's model and made an improvement to it. Group C also took group A's model and changed it to apply to a different cell type. Group D then combined the improvements of group B with the change of cell type by group C. Group E then fixed a different issue in group A's model. A user of a revision history unaware repository will see 5 models - from groups A, B, C, D, and E, with little information in a machine readable form about the relationship between them. In this case, there is the potential to update the model from group D with the improvements from group E. A machine readable revision history makes it possible to establish the relationship between group D's and group E's models, and, possibly, depending on the exact location of the changes, automatically merge the changes from the models to make a new model. Without this machine readable revision history, merging would be a manual process, and the easiest way to update group D's model would be to read E's paper and add any improvements to group D's model.

The question of how revision history is maintained for computer software has an extensive history. SCCS (source code control system) [13] and RCS (revision control system) [14] were early solutions. These systems treat each file as a separate module, with independent version history, and treat revision history as a tree. Every revision of a file (after the initial version) has a single parent revision. An RCS repository has a trunk, to which all new revisions are generally committed, and the possibility of branches, in which derivatives of an older revision on the trunk (or another branch) are created. Selected changes made to the trunk can be merged into branches, and vice versa. However, the fact that this merge occurred is not recorded by the RCS software in any machine readable form (so as to preserve the tree structure of the revision history), meaning that part of the revision history can be lost (especially when multiple revisions are merged at the same time). This means that if, for example, changes made on a trunk are merged into a branch, a later attempt to merge all changes on the branch back into the trunk will result in conflicts, as the branch already includes some of the changes from the trunk. When the merge is successful, all changes on the branch will appear, in the revision history of the trunk, as a single change.

The Concurrent Versioning System (CVS) [15] extends RCS to work over the network, which allows multiple developers to work on the same software package on separate computers. However, the underlying process of storing the revision history for each file separately, and in a tree-like fashion remains. These concepts are further extended in Subversion [16]. Subversion retains the requirement for revision histories to form a tree. However, entire hierarchies of directories and files are versioned as a single module, with atomic changes made across the entire hierarchy.

More recently, there has been a major shift towards distributed version control systems (DVCSs). Darcs [17], Pastwatch [18], Monotone [19], Git [20], and Mercurial [21] are all examples of such systems. These systems allow every user to have a local repository to which they can commit changes, combined with the ability to push and pull sets of changes between repositories. In order to achieve this, the revision history is allowed to form a directed acyclic graph (DAG), rather than being limited to a tree structure. This means that two or more people can make and commit changes from the same starting version of a file. When these people want to push their changes to the same centralised repository, their changes need to be reconciled. In CVS or Subversion, this would be achieved by requiring users to update and merge before they commit. However, in DVCSs, the commit has already been made, so instead, a new revision representing the merge, with two parents, is created (for example, the parents might be the changes of the first person to push, and the changes of the second person wanting to push).

The same properties which make DVCSs useful for distributed development also make it useful for developing different versions of software under version control. Unlike in earlier centralised VCSs, it is possible to merge back and forwards between a branch as many times as required without conflict, as merges are revision history aware, and so no attempt is made to merge changes which have already been merged. While DVCSs are most widely used for maintaining software, there is no reason why their utility should be limited to software. Computational models represented in a markup language have features in common with both computer software, and with marked up text documents (both of which can be represented in a DVCS). However, prior to the work described here, we are not aware of any other previous use of a DVCS for mathematical models of a biological system.

Saffrey and Orton [22] discussed the differences between the version control requirements of software and models, and proposed the use of XML patches so that different combinations of changes to models can be applied. They adopted this solution because it allowed a wide range of variations of a base model to be created in semi-automated way. The requirement in traditional VCSs that the revision history must form a tree makes it difficult to maintain models made by applying different combinations of changes. DVCSs solve this problem because they allow one revision to have multiple parents, making it possible to maintain branches produced by the application of different combinations of changes.

This paper presents our research into how DVCSs can be used in model repositories to better track the revision history of computational models of biological systems.

## Methods

### The application of DVCSs to computational models

We will focus here on CellML models. CellML 1.1 models can consist of one or more files. Each model has a top-level CellML file, which can import files containing different CellML models, and use components from them as sub-models. In some cases, the top-level model will merely finalise the model by connecting cell-type specific parameters, or a particular experimental stimulation protocol, to the remainder of the model [23], and so there may be several alternative top-level models, with the revision history being recorded across several files as a single module.

However, in other cases, a model may import a model of a functional module, such as a particular ion channel [24], which has great generality. The ion channel model might be re-used as part of many different larger models. It would not make sense for all possible larger models which might use the ion channel to be included in the same directory. Instead, what is required is the ability for each complex model to include, by reference, a particular version of the model to be imported.

### Supporting a DVCS in PMR

We adapted the existing PMR software (to create PMR2) so that all models put into the model repository are stored in a Mercurial repository. We refer to each local Mercurial repository as a workspace. Unrelated models would usually be stored in different Mercurial repositories. However, where several very closely related models, each of which shares common parts, are created by the same author, they may be stored in the same workspace.

Workspaces can contain other files alongside the model files. For example, they can include SVG diagrams or raster images which aid in understanding the model. The repository can also include tool specific data, such as OpenCell session files http://www.opencell.org/, which describe how to display the model to users. These files can be arranged in an arbitrary structure of directories. PMR2 can be used for non-CellML models.

Authorised users are able to push changes to their Mercurial workspace. For models which are made public, anyone can pull changes from the workspace through Mercurial, and can also browse different revisions in the workspace, or download all model files in the workspace as of a particular revision, using a web interface. Users can pull a workspace which was created and made public by another user, make and commit a series of local improvements to the model, and once authorised, push these changes back to a new workspace in the model repository. The new workspace will contain all the changes that were put into the original workspace. It is then possible for the author of the original workspace to pull changes back into their own workspace.

### Embedded workspaces: dealing with common sub-models

Using Mercurial, it is possible to nest one workspace inside another (referred to as embedded workspaces). This allows a user to include a common sub-model, such as an ion channel model produced by someone else, in a model, while maintaining control of exactly which revision of the submodel is being used. This enables the model to be described using relative URIs.

There are several reasons why this is better than using an absolute URI in the importing model to reference the model repository:

• It greatly simplifies the process of updating the sub-model, as this can be done through an ordinary Mercurial pull and update of the submodule, rather than requiring that the importing model be edited and the URIs be changed.

• It means that it is simple to obtain the complete model and revision history, for use offline.

• It greatly simplifies the workflow when the same user is creating both the sub-model and the importing model. Instead of needing to completely specify the submodel, push it to the workspace on the repository, obtain the URI, and then set up the importing model, the modeller can instead work on both models in parallel, and push the top-level and sub-models to the repository later when they are ready.

### Exposures

Not every change committed or pushed to a workspace is necessarily an overall improvement to the usefulness of a model, compared to the previous revision of the model. For example, a change might correct one problem in a model, but due to fudge factors or parameters which have not yet been refit, actually make the results of the model less accurate. In addition, a change might improve a model from a numerical sense, but the previous revision might have been through a careful peer-review process, while the latest revision is the unchecked work of a single person.

For these reasons at least, it is not true that the latest revision in a workspace is always the one that should be advertised to casual users of the model repository. The concept of exposure was therefore added. This is a reference to a particular revision in a workspace, with some associated documentation. The exposure acts as the link to databases such as MEDLINE (http://www.ncbi.nlm.nih.gov/pubmed/; using the PubMed ID), which identifies a published paper, and the particular revision of a workspace. It also holds information on the results of any validation carried out by the repository curators.

### Tracking revision history in practice

PMR2 was deployed to the CellML Model Repository to replace PMR in June 2009. In order to achieve this, all models in the existing CellML Model Repository (which simply had different versions with ordinal revision numbers) were migrated into the DVCS using a script which added each version as a sequential revision. The date at which each revision was added was preserved as the date of addition.

We predicted that moving to the DVCS based repository would increase the amount of revision history information (as measured by the number of revisions) for several reasons.

Firstly, model authors and curators can make a series of changes, some of which may be a conceptual improvement on the model, but which actually make the model less usable until other issues are resolved. Under the previous non-DVCS repository, revisions would only be put up by curators when they were ready for the public to use, losing information on the intermediate steps taken (which may involve several model authors and curators) to get to that state.

The previous repository also allowed curators to retrospectively replace revisions in cases of minor changes, again losing part of the revision history. In addition, the previous repository did not version figures and other supplemental data alongside models. Under a DVCS based repository, curators and model authors can directly push and pull changes to and from each other. They can also share a model which is not yet ready for publication through a workspace in the repository, and create an exposure for the model when they are ready. Alternatively, they can share a model by pushing to or pulling from their respective local repositories directory, and all revisions made will be added to the repository when the changes are pushed to the repository.

To test the above prediction, we analysed the model repository as of the 28th of April, 2010 (310 days after the deployment of PMR2). There were, at that point, 516 workspaces in the repository. There were 2,273 different revisions in the repository, of which 1,426 were migrated from the previous repository.

**Figure 2:**
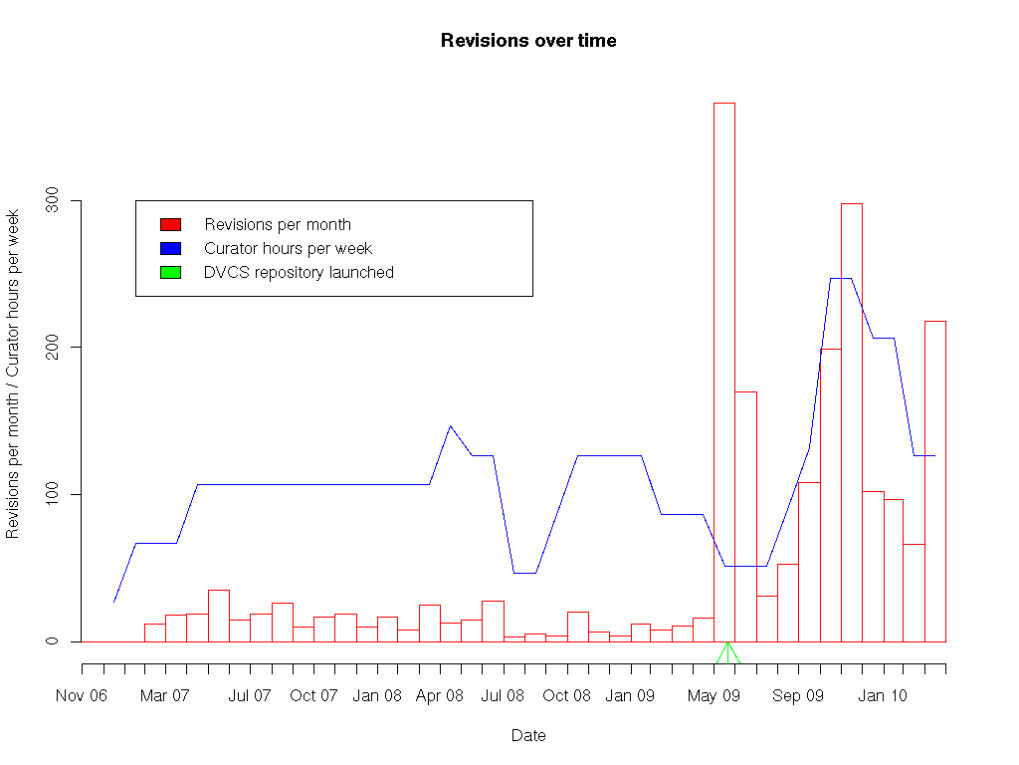
**Revisions over time**. This figure shows the total number of revisions committed to models in the repository each month. Only revisions which were pushed to the repository by the 28th of April, 2010 are shown. The graph also shows an estimate of the total number of hours of model curation work contracted per week, and includes an arrow to indicate the point in time when the repository was switched over to use DVCS-based workspaces.

Figure 2 shows a time series of the number of revisions made across the entire repository per month. One major expected explanatory variable, the number of hours curators were contracted to work per week, is also shown on the graph. Data on curator hours worked per week were obtained by asking the curators. The values plotted are averages for the month. The spike in June 2009 is partly due to automated changes made (over the course of less than an hour) to all models as part of the repository migration process. We excluded the June 2009 data point from further analysis, as these automated changes were deferred until the deployment of the repository, and so could bias the analysis.

**Figure 3:**
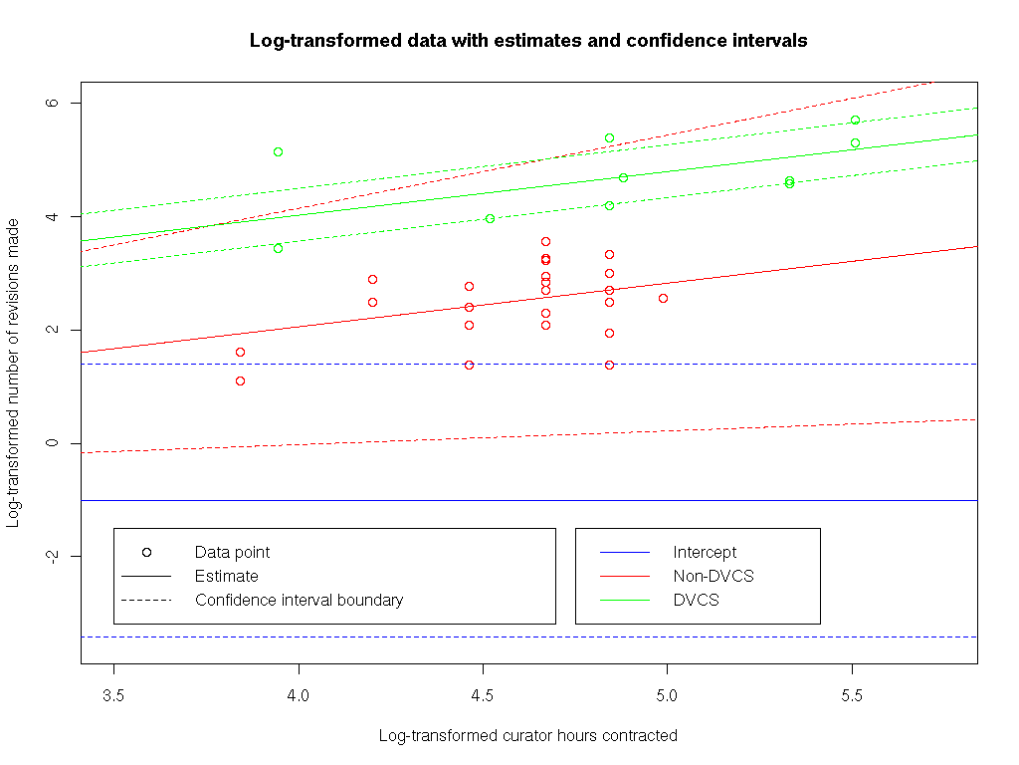
**A visualisation of the statistical model**. This figure shows all monthly data points on log-transformed axes for the number of contracted curator hours (**c*'***) and the number of revisions made (**r*'***), colour-coded to distinguish between points from before and after the switch to DVCS-based workspaces. Overlaid on this figure is the estimator $\hat{\alpha }$, shown as a horizontal intercept, the estimator $\hat{\gamma }$, shown as the red line $\hat{\alpha }+\hat{\gamma }c'$, and $\hat{\beta }$, shown as the green line $\hat{\alpha }+\hat{\beta }+\hat{\gamma }c^{'}$. In addition, dashed lines corresponding to the boundaries of the 95% confidence interval for each parameter is shown (note that each confidence interval is shown separately, and so, for example, the dashed green lines do not include uncertainty about *γ*).

We fitted a generalised linear model [25] that the log number of revisions (from February 2007 - April 2010) is explained by the log number of hours that curators are contracted to work, and the factor of whether the repository used DVCS-based workspaces. We log-transformed the data, as we believe that it makes more sense for the factors affecting the rate of revisions to be multiplicative rather than additive. In other words, we fitted the model **r*' ***= *α *+ *β***d **+ *γ***c*'***, where **r*' ***is the log-transformed vector of revisions in each month, **d **is 0 if DVCS-based workspaces had not been deployed, and 1 if they had, and **c*' ***is the log-transformed number of contracted curator hours. There is very strong evidence that *β *is non-zero (*p *< < 0.001), and evidence that *γ *is non-zero (*p *= 0.00509). The unbiased parameter estimates are $\hat{\alpha }=-1.01$, $\hat{\beta }=1.97$, and $\hat{\gamma }=0.769$. In other words, after the log-transforms are simplified out, $\hat{r}=0.363c^{0.769}d^{'}$, where $\hat{r}$ is the estimator of the number of revisions per month, *c *is the number of contracted curator hours, and *d' *is 1 if the DVCS repository has not been deployed, and 7.16 otherwise. The 95% confidence interval for exp(*β*) is (4.51, 11.4). The data, and the fit of the model to it, is visualised in Figure 3.

This analysis shows that there is good evidence that the ratio of number of revisions per week $\frac{\text{after}}{\text{before}}$ switching to a DVCS was somewhere between 4.51 and 11.4.

### Model authors and curation

In many existing repositories, access to create revisions of a model is restricted to authorised curators. This is done so that curators can ensure that models that are to be made public meet the standards of the repository. However, this also has the side effect that model authors cannot create revisions except by submitting their changes to the curators, and usually means that a record of the revision is only created when a model is accepted by the curator (which may require a number of changes).

DVCSs work by recording changes locally, and only later pushing them to another repository. This means that model authors can make revisions and store them on their own computer without the need to be granted access by the repository curators. It is the act of pushing these changes into the central repository that requires access to be granted. In PMR2, model authors are given access to their own workspace, and it is only the final step of creating an exposure of a particular revision on the repository website which is restricted to authorised curators. This approach allows the quality of exposed models to be maintained, while not preventing model authors from creating as many revisions as required to accurately describe the revision history of their model.

### Presenting models with revision history to casual users

An important part of a model repository is the ability to present the information it contains to casual users. Model repositories based on PMR2 are accessible using a standard web browser, presenting exposures and workspaces to casual users.

**Figure 4:**
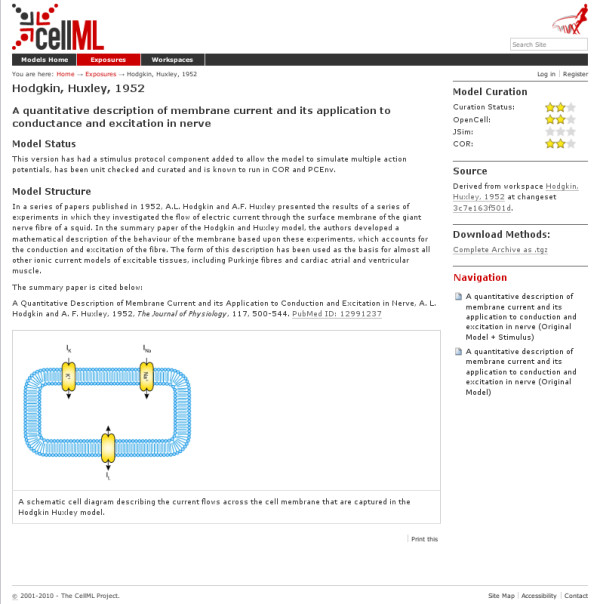
**PMR2 user interface - exposure view**. This figure shows the web-based user interface for viewing exposures. The model shown is a CellML representation of the Hodgkin & Huxley 1952 model. The view provides both information on the version the exposure relates to, and a link back to the workspace for revision history.

As is shown in Figure 4, the web rendering of an exposure of a model (in this case [26]) includes a rendering of structured machine-readable metadata about the model (for example, the display of how well the model works in different tools, to the right), unstructured (human-readable) metadata from the model, and a section on the source of this model (the workspace, and the revision shown in the exposure). This section also provides a hyperlink to the workspace information.

**Figure 5:**
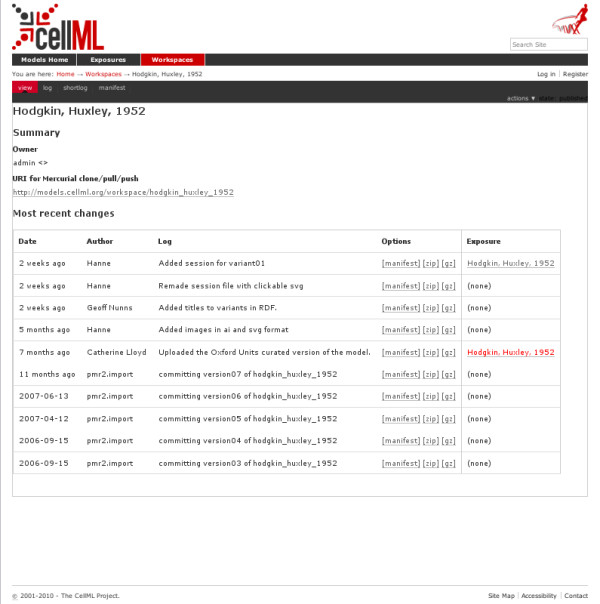
**PMR2 user interface - workspace summary view**. This figure shows the web-based user interface for viewing workspaces. The model shown is a CellML representation of the Hodgkin & Huxley 1952 model. The view shows the history of the model leading up to the current version, in reverse chronological order.

There are several views of the information in the workspace available, but the first view a user sees after viewing the workspace from the exposure is shown in Figure 5. This view displays the sequence of changes which were made, and the author of the changes, in the order they occurred. For revisions where one exists, it also provides a hyperlink to the corresponding exposure.

## Conclusions

Maintaining accurate records of the revision history of computational models plays an important role in understanding the origin and justification for certain parts of a model, and it also allows for more accurate automated merging to facilitate scientific collaboration when building models.

Existing DVCSs can successfully be incorporated as part of a model repository, and this approach allows much better change records to be kept when compared to storing a linear series of model files. It accommodates models developed in parallel by different people, with features from each being merged, and ensures that all changes are recorded in the revision history. By separating the concept of a revision from the concept of an exposure of a model, and by applying access and quality control restrictions when creating an exposure, rather than when recording a revision, model authors and repository curators become free to provide an entry in the revision history for every change made.

In practice, deploying a DVCS to replace a linear series of CellML model files has led to a significant increase in the number of revisions being captured in a repository.

## Availability and requirements

We have included the raw data (in the form of a text file with the times and dates of all commits), giving revisions by date as a plain text list of timestamps, as additional file 1. In addition, we have provided two scripts for generating updated data as supplementary files: additional file 2, a Python script which can be used to download the latest version of the entire CellML model repository, with all revision history, and additional file 3, a shell script which will generate the list of revision dates (*i.e*. additional file 1) from a downloaded copy of the model repository.

The R script used to generate the statistics and figures in this paper from additional file 1, the list of revision dates, is also included as additional file 4.

In addition, we note that we have made use of a range of Free/Open Source software for this analysis. The R software is available at http://www.r-project.org/. The PMR and PMR2 software packages are also Free/Open Source, and instructions for obtaining and configuring the software is available at http://www.cellml.org/tools/pmr/installation.

## List of abbreviations

**CellML: **An XML-based markup language for mathematical models; **CVS: **Concurrent Versioning System (a specific VCS); **DVCS: **Distributed Version Control System; **PMR2: **Physiome Model Repository 2 (software for creating model repositories based on DVCS); **RCS: **Revision Control System (a specific VCS); **SBML: **Systems Biology Markup Language (an XML-based markup language for systems biology models); **URI: **Uniform Resource Indicator; **VCS: **Version Control System; **XML: **Extensible Markup Language

## Authors' contributions

AKM first suggested the use of a DVCS for recording the revision history of CellML models, wrote the initial manuscript draft, and carried out the statistical analysis of the number of revisions. TY wrote the PMR2 software. All authors provided feedback on the concepts underlying the use of a DVCS to build a model repository, and on this manuscript. All authors read and approved the final manuscript.
